# Evaluating the impact of shift work length and time of day on musculoskeletal disorders among nursing assistants in long-term care

**DOI:** 10.1016/j.hfh.2025.100094

**Published:** 2025-06

**Authors:** Ryan L. Bellacov, Kermit G. Davis, Chunhui He

**Affiliations:** College of Medicine, University of Cincinnati, Cincinnati, OH, USA

**Keywords:** Shift work, Extended work shift, Long-term healthcare, Certified nursing aide

## Abstract

**AIM:**

This study aimed to investigate differences in activity and body movements for the different 8-hour shifts and extended 12-hour shifts at a long-term healthcare facility. The secondary aim will focus on determining the impact of shift work time of day on musculoskeletal injuries causing days off.

**Background:**

Nursing assistants have heavy physical workloads, which results in high turnover and musculoskeletal pain.

**Methods:**

The research team observed 240 shifts for 54 nursing assistants in five long-term healthcare facilities. The researchers completed direct observations of posture through the utilization of an ergonomic evaluation tool using the Rapid Entire Body Assessment using a random sampling (equivalent of 12 times per hour). An activity monitor continuously captured the time spent sitting, standing, and walking, along with the associated energy expenditure while working.

**Results:**

The most significant results were in physical activity, with an 8-hour day percent of total time walking being double the amount at 48.4% compared to night shifts at 23.1% (total steps per hour: 1704 compared to 763, respectively). REBA scores for the upper body on the day shift are more ergonomically challenging (risk rating: 7 to 5, respectively). The 12-hour shifts negatively impact on the lower back and shoulder, causing injuries in long-term care.

**Conclusion:**

Overall, an 8-hour day had the most impact on energy expenditure. In addition, 8-hour shifts had a higher incidence of lower back pain, resulting in the most frequent loss of work. The incidence of loss of workdays affects employee well-being, productivity, and medical associated costs. Nearly 20% of the nurse assistants’ observations had poor posture with medium to high risk.

**Implications:**

Healthcare managers could help the physical strain that nursing assistants endure by targeting footwear, poor posture, and fatigue. While the higher workload in this study did not equate to higher musculoskeletal pain, the complexity of the normal work tasks likely resulted in complex demands on the nursing assistants.

## 1. Introduction

Approximately 20% of workers in the United States work non-standard schedules such as rotating and night shifts, with healthcare being one of the shift work industries (US Congress, 1991; Gerstel & Clawson, 2018; Gourevitch et al., 2023). Shift work usually means working outside the normal daylight hours (8am—5pm) or more than 8 h at a time (Riedy et al., 2021). This study will focus on abnormal shift lengths and time of day compared to standard 8-hour shifts. Many studies have found a negative impact of shift work on health and well-being, yet gaps exist in how this affects musculoskeletal injuries (Boivin et al., 2022; Mélan & Cascino, 2022; Puttonen et al., 2022).

The need for 24-hour continuous service to meet healthcare requirements makes shift work necessary. Even though the health effects of the 12-hour shift on caregivers are not conclusive, many hospitals and long-term care facilities have implemented 12-hour shifts. The history of hospitals adopting 12-hour work shifts is characterized by a complex interplay of workforce management strategies focused on labor short-ages several decades ago and the benefits of fewer transition issues for two shifts (U.S. Bureau of Labor Statistics, 2020; Koy et al., 2022). Extended shifts are likely to continue as many employees as possible like the flexibility of fewer working days (e.g., 3 days of 12 h) and healthcare facilities’ perceptions of less critical transitions between nurses. However, there are negative considerations about patient care and the safety implications of longer shifts related to increased fatigue and more prevalent accidents (Grewal & Holt, 2022; Patterson et al., 2022).

According to Ohio Administrative Code 3701–17–08, 2.5 h of direct care per resident daily is like most states. Nursing assistants’ primary task of caring for people often responsible for direct care is a very demanding job with unknown complexity (Li et al., 2024; Mazzoli Smith et al., 2023). In addition, the complex demands of long-term care have frequently led to higher-than-average injuries and musculoskeletal pain and disorders (Bakker et al., 2009; Davis & Kotowski, 2015; Denham et al., 2023). National data has shown a work-related injury or illness requiring medical treatment or lost work was 8.8 per 100 among long-term care facility workers (Bureau of Labor Statistics, 2021). Among those injuries in healthcare, musculoskeletal disorders (MSDs) are the majority, resulting in substantial costs, especially low back pain Davis and Kotowski (2015); Ji et al. (2023); Kugler et al. (2024); Matsumoto et al. (2016). Furthermore, Long and Silverstein (2013)) reported that long-term care facilities were the #1 sub-industry in the number of musculoskeletal disorders for over a decade and were #23 in costs with 5.14 per 100 FTE (Long et al., 2013; Silverstein et al., 2002).

The musculoskeletal disorders injury rate for nursing assistants was double that of a higher-level healthcare provider (8.8 vs. 4.1 per 100 FTE) (Kotejoshyer et al., 2019). Randall and associates (2009) found that nursing assistants were more than four times more likely to be injured when lifting bariatric patients than registered nurses (62% to 15%) (Randall et al., 2009). During the professional career of nursing, a lifetime low back pain prevalence of 84.7% over a year has been reported (Bruno et al., 2015; Gilchrist & Pokorná, 2021; Jahani et al., 2022). In some healthcare studies, this prevalence of lower back pain among nurses might be explained by the compressive loads on the spine when performing patient-handling tasks (Marras et al., 1999). Unfortunately, the data shows nursing assistants suffer from one of the highest rates of low back injuries.

Although data has shown that nursing personnel experience a high incidence rate of musculoskeletal symptoms, the role of shift work in developing these problems has yet to be fully understood (Farooq & Chaikumarn, 2023; Trinkoff et al., 2006). Research has shown that abnormal times of day shift work can cause many adverse effects on health-related issues, particularly disturbed sleep and fatigue (Chen et al., 2011). Further, it appears that the long-term effects of shift work are more associated with chronic diseases such as coronary heart disease (Bortkiewicz, 2023).

The *objective* of the proposed study was to quantify the physical demands among nursing assistants working on different shift work schedules. The study divided shift work schedules by comparing the length and time of day. The primary aim focused on activity and ergonomic evaluations correlated to musculoskeletal discomfort and injuries among nursing assistants working on standard 8-hour vs. 12-hour shifts in long-term care facilities with nursing assistants. The secondary aim will investigate the time of day’s influence on work activity, ergonomic evaluations, and injury rates.

## 2. Methods

This study examined relationships between activPAL^™^-determined physical activity and ergonomic assessments with musculoskeletal injuries. The study focused on nursing assistants in medium-to-large long-term care facilities. The current study focused on the impact of shift length on physiological indicators and musculoskeletal issues while a concurrent study investigated psychosocial factors for participants.

### 2.1. Subjects

Fifty-four (54) nursing assistants who worked in one of the five long-term care facilities of similar size (80–100 beds) participated in the study. These facilities were in the Cincinnati metropolitan area, representing typical long-term facilities in the Midwest. The subjects were permanent employees without physical restriction and had worked with the subject’s employer for at least three months. All nursing assistants had experience as healthcare givers at the current facility or another facility. Self-reported demographic information from subjects is shown in Table 1.

At the end of the data collection, the participants were paid $50. Before recruitment of long-term care nursing assistants, researchers requested facilities direct recruitment to the general population, restricting specialized care. The institutional research ethics committee obtained ethical approval. All participants provided written informed consent before participation.

### 2.2. Shift work protocol

The study was cross-sectional, where the researchers observed nursing assistants in long-term care facilities performing daily tasks throughout the shift. The physical demands and musculoskeletal discomfort/injuries were documented for nursing assistants who worked on 8-hour and 12-hour shifts and three categories for the time of day. The sample population worked one of the following shifts: 8-hr-day (7:00am to 3:00pm), 8-hr-evening (3:00pm to 11:00pm), 8-hr-night (11:00pm to 7:00am), 12-hr-day (7:00am to 7:00pm) and 12-hr-night (7:00pm to 7:00am). This study focuses on shift length and time of day by comparing physical activities, postures, and the reported injuries among nursing assistants.

### 2.3. Outcome measurements

#### 2.3.1. Physical and posture measurements

Typically, physical demands are assessed by analyzing posture, movement, and peak or cumulative force achieved in this study (Burdorf & van der Beek, 1999; Kadikon & Rahman, 2016). The ActivPAL monitor quantified total body physical activity across two shifts for selected nursing assistants (ActivPAL3, PAL Technologies, Scotland, UK). A small activity monitor (ActivPAL) was attached to the thigh of the nursing aide. The use of physical activity monitors to quantify sitting, standing, and walking was accurate (Aminian & Hinckson, 2012; Wafa et al., 2017; Wu et al., 2022), and widely utilized for quantification (Pickford et al., 2019). The ActivPAL monitor records the number of steps, time spent sitting, standing, and walking, and estimated energy expenditure. The activPAL monitor scores activity using an accelerometer to detect body posture and movements, distinguishing between sitting, standing, and walking. It processes acceleration data to quantify time spent in various activities and the number of steps taken.

**Fig. 1.**
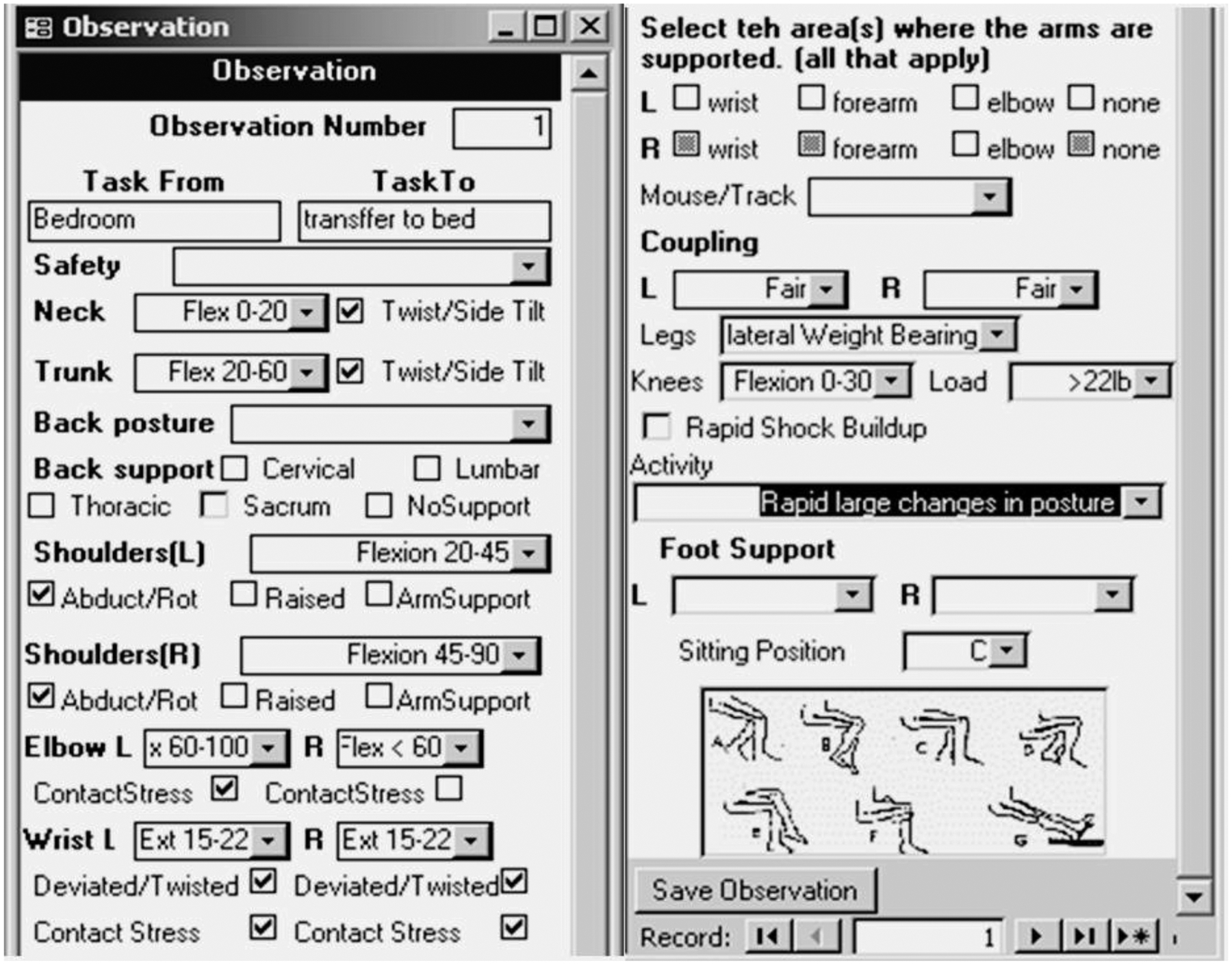
Screen caption of the PDA Rapid Entire Body Assessment (REBA) checklist designed collect data in the field.

In this study, the posture demands were classified by direct observation using a checklist developed based on the Rapid Entire Body Assessment (REBA) (Hignett & McAtamney, 2000; Wibowo & Mawadati, 2021), which previous researchers utilized to evaluate postural demands in the workplace (Torres & Vina, 2012; Wibowo & Mawadati, 2021). The REBA program was designed by Oliver-Hernandez and associates (2023) for field studies and validated in hospital settings (Oliver-Hernández et al., 2023). The program calculated an upper body index that identified the postural demands on the arms, shoulders, hands, wrists, and neck and a lower body index that identified the postural demands on the lower back, upper leg, lower leg, and ankle/-foot (Oliver-Hernández et al., 2023). The REBA tool helps identify ergonomic risks quickly. For example, the posture of the neck is scored based on the angle of deviation from a neutral position, with scores as follows: 1 point for a neutral or slightly deviated angle (0–10°), 2 points for a moderate deviation (11–30°), and 3 points for an extreme deviation (>30°). Higher scores indicate an increased risk of musculoskeletal disorders associated with poor neck posture (Gerstel & Clawson, 2018). The PDA checklist was designed for field studies and has been previously utilized in hospital settings (Fig. 1) (Riedy et al., 2021).

#### 2.3.2. Sampling rate

At least one observation was made every 20 mins per subject that Microsoft Excel randomly assigned with the feature of “generate random numbers within a range.” Posture observations were recorded in 5-minute increments. Random sampling intentionally avoided bias related to specific times of day or routine activities. This random sampling method provides a more representative picture of overall posture than if observations had been taken at fixed intervals, such as every 5 mins, because it captures a broader range of times and circumstances.

### 2.4. Loss days from work

This study included a series of three questionnaires about work loss days per body region used to define work impairment. The first question asked the subject to estimate over the past year. “How many days out of the past year were you unable to work or carry out your normal activities?” The subject had three responses on work loss days to give either zero, less than a week, or more than a week. Among those who reported such days, subjects were asked about pain areas.

### 2.5. Data and statistical analysis

The independent variables were shift parameters: shift schedule and duration (8-hr day, 8-hr evening, 8-hr night, 12-hr day, and 12-hr night). The dependent variables for physical demands were posture data and physical activity. Direct observation data obtained from the entire shift was used to quantify the physical demands required of nursing assistants. To eliminate the duration effects, the data collected by ActivPAL was calculated using an hourly rate or percentage of the shift duration. Each posture category was coded and set up as an event. Hourly frequencies were then calculated by summing up the events for specific postures for that hour. The dependent variables for physical demands were postured data (UB and LB indices and frequency of different joint angles). A one-way analysis of variance (ANOVA) was conducted to identify if there are any statistically significant differences in posture scores across multiple work shifts. If ANOVA found significant differences, a post hoc Tukey test was then used to pinpoint which specific shifts differed from each other. The one-way analysis of variance (ANOVA) for activity with activPAL was similar, looking for statistically significant differences in sitting/lying time, standing time, walking time, step count, sit-to-stand transitions, cadence (steps per minute), and duration of each activity compared to each shift type.

To assess the relationship between shift work and discomfort, the level of current discomfort in multiple body regions was utilized as dependent variables, which included the lower back, hips, legs/feet, shoulders, hands/wrists, and neck. These data were obtained from the symptom survey. The independent variable was again the shift of the nursing assistants. A summary of the statistical analysis is given in Table 2.

## 3. Results

While the study does encompass five distinct long-term care facilities in the Cincinnati area, many characteristics were similar, such as most rooms had **two residences per room**, resident characteristics were more independent, not having the complexity of Alzheimer’s disease, the staff-to-resident ratio was the same, conforming to regulations, and tools to ease physical work the same.

### 3.1. Physical activity comparison

The results from ActivPAL on physical activities among the nursing assistants (See Table 3) indicated that the day shift had more energy consumption than the night shift, with the 8-hr-day shift having the highest physical workload. The nursing assistants on the 8-hr-day shift were on their feet more, which ActivPAL showed workers on an 8-hr shift were on their feet 90% of the time. In addition, the 12-hr-night shift had the lowest energy expenditure, with the lowest amount of walking but the highest prolonged standing.

### 3.2. Posture comparison

#### 3.2.1. Postural

**Fig. 2.**
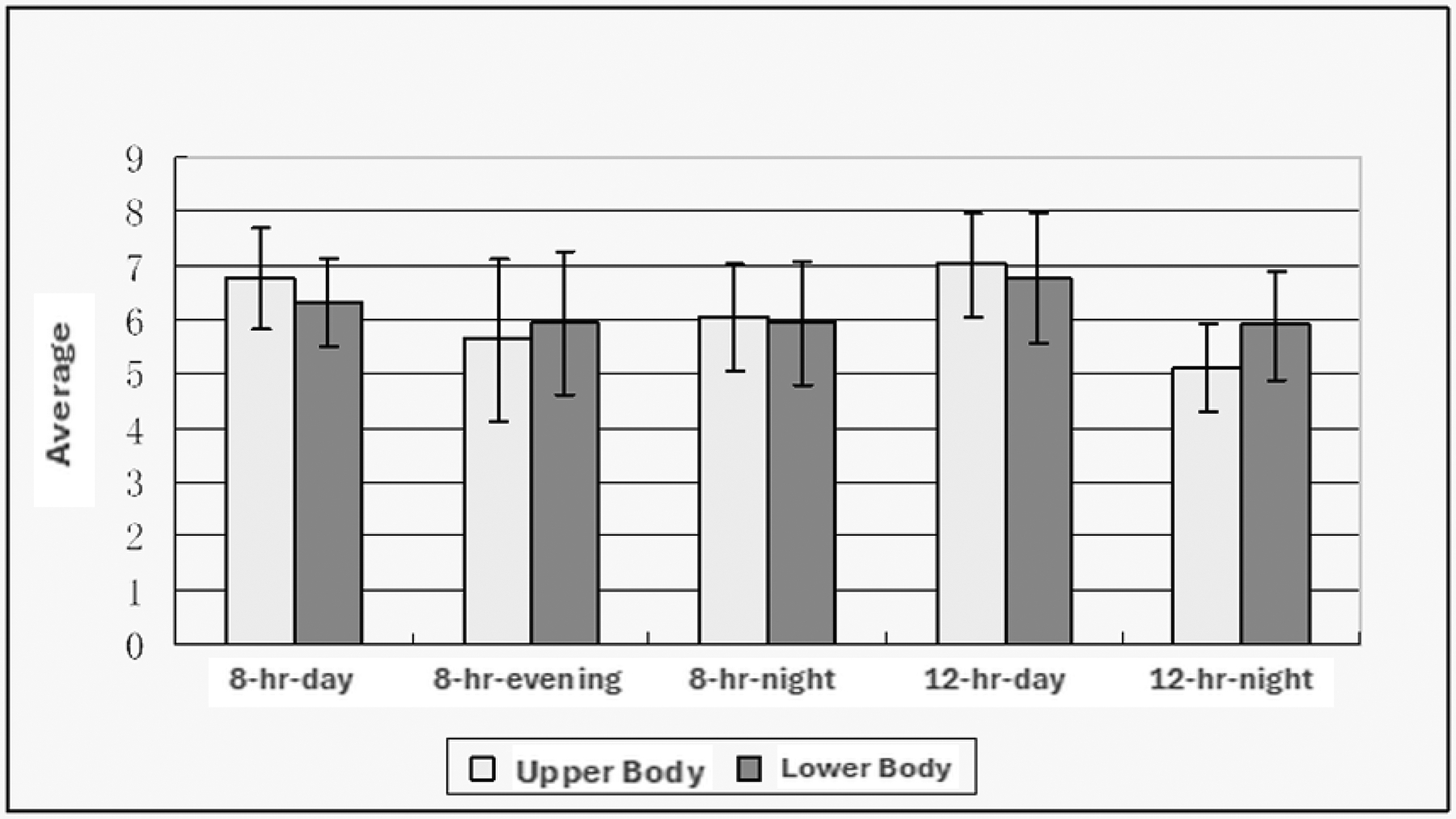
Average Rapid Entire Body Assessment (REBA scores range from 1—low risk to 12—high risk) score for upper and lower body posture scores of nursing assistants for each shift (8-hr-day, 8-hr-evening, 8-hr-night, 12-hr-day, and 12-hr-night).

The REBA checklist results indicated a significant difference (*p* < 0.05) among the shift workers for upper extremities. The awkward posture of the upper extremity appeared to be the driving factor for the higher score. As seen in Fig. 2, the upper body posture score for nursing assistants working the 8-hr shifts was about 40% greater than the 12-hr shifts. A high REBA score indicates awkward postures, repetitive movements, or excessive force exertion during nursing assistants’ tasks of the arms. However, the lower body score showed no significant difference among the nursing assistants. Still, the trend was that the 8-hr-evening shift had the highest scores for both upper and lower body postures.

Knee motion during day shifts appeared to be driven by walking and standing during the shifts. The night shifts of 8-hr and 12-hr shifts had the higher counts/hour for poor knee posture, most of the time within the range of 30° to 60°. The 8-hr shifts had more elbow flexion activity within 20° than other shifts. There was no significant difference between 8-hr-evening (5.42 times/hr.), 8-hr-night (5.90 times/hr.), and 12-hr-night shifts (5.01 times/hr.) for left elbow flexion between 60° to 100°. Although the left shoulder had a lower rate of joint activity than the right shoulder, the 8-hr shifts had more shoulder flexion activity between 45° and 90° than other shifts. The 12-hr shifts had similar observations as the 8-hr shifts on elbows and wrists, more corresponding to the time of day.

When comparing wrist postures, the 8-hr shifts fell into 0° to 15° flexion/extension most often and had significantly greater counts (*p* < 0.001) than the other shifts, with 44% more than the 12-hr shifts. In the 15° to 22° category, the wrist posture of the 12-hr-day shift ranked highest, which was 71% more than the 8-hr-day shift, 28% more than the 8-hr-evening shift, 25% more than the 8-hr-night shift and 12% more than 12-hr-night shift (*p* < 0.03).

Trunk postures further support more walking and standing for the day shifts and more sitting for the night shifts. Poor trunk postures were relatively infrequent (less than three times per hour), potentially indicating good body mechanics while performing their tasks. The trunk position showed that 8-hr-day shift workers performed tasks with trunk upright more than other shifts. The difference was that the 8-hr-night and 12-hr-night shifts had less trunk flexion and extension. In addition, 8-hr-night and 12-hr-night shift nursing assistants sat more than day workers.

No difference was found for severe back postures, such as trunk flexion above 60°. However, the trend demonstrated that the 8-hr-night shift was the worst among the shifts. However, when combined with the severe posture in the categories for each joint (as in Table 4), shift differences were identified for the neck, elbows, and wrists. The 8-hr shifts had a greater rate of awkward postures for the neck, which was higher than the 12-hr-day and 12-hr-night shifts by 80% and 25%, respectively. In addition, the 8-hr shifts had more severe postures for shoulders and elbows. Compared with other shifts, the 8-hr-night shift had more elbow flexion of less than 60° and extension of more than 100° on the right side than the other 8-hr shifts. Similarly, for the right shoulder flexion of more than 45° and extension of more than 20°, the 8-hr-evening shift had a higher rate than the other 8-hr shifts and the 12-hr shifts with the greatest amount. The 8-hr-day shift had the least awkward postures. The severe postures for the trunk showed no significant difference.

#### 3.2.2. Lost work days injuries and body discomfort symptoms

As reported in the survey, more nursing assistants lost workdays due to lower back pain during 8-hr shifts, with the lowest number for 12-hr shifts. The 8-hr shifts also had more loss of workdays for more body regions than 12-hr shifts, with only 10% of nursing assistants on 12-hr shifts losing 1–2 weeks of workdays due to knee pain.

**Fig. 3.**
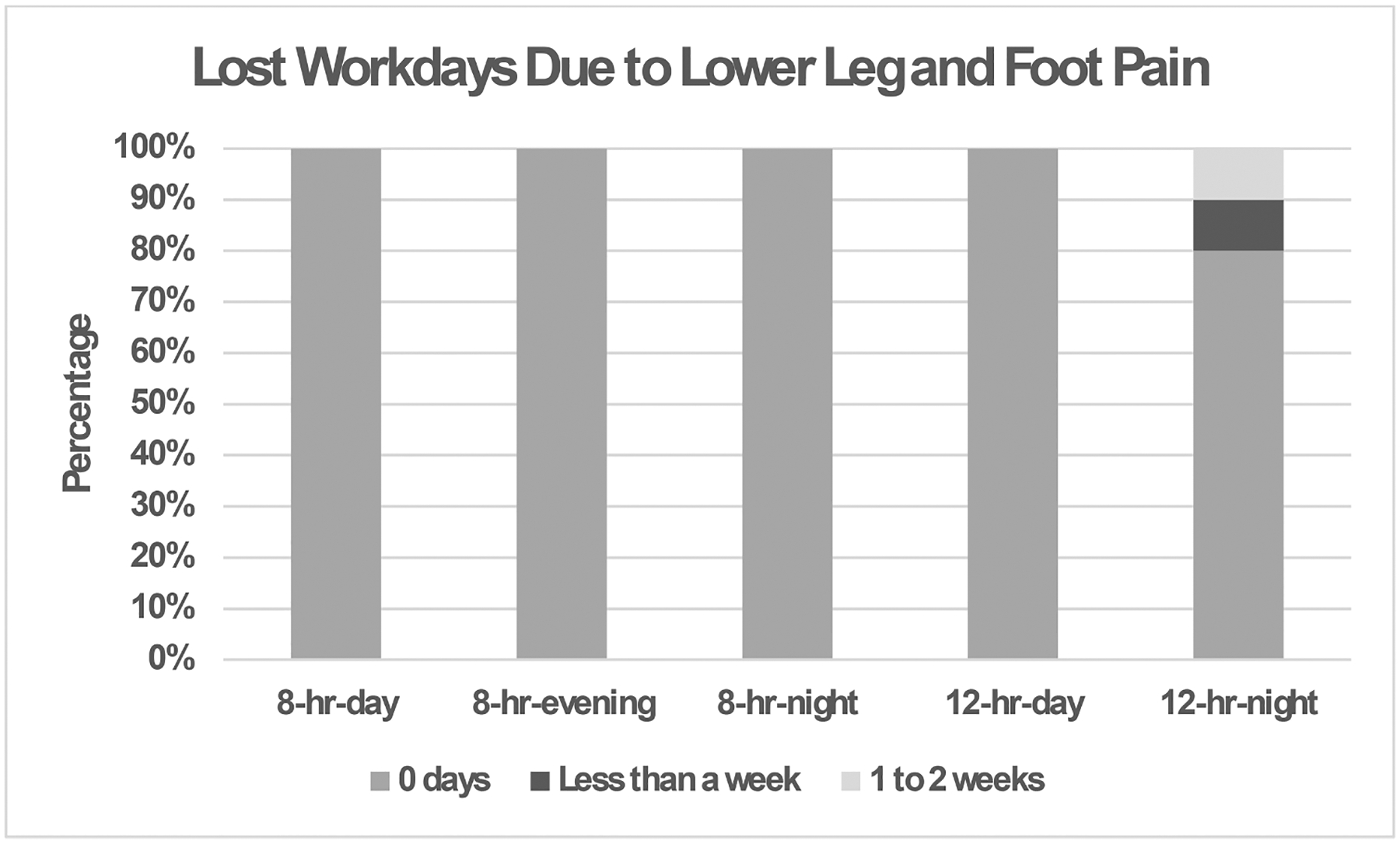
Percentage of nursing assistants with lost workdays due to lower leg and foot pain (*p* = 0.03) in the past year for each shift (8-hr-day, 8-hr-evening, 8-hr-night, 12-hr-day, and 12-hr-night).

The results showed a significant difference in lost workdays due to ankle and foot pain for each shift in the past year. The 8-hr-night shift had the most responses due to ankle and foot pain in the past year, with 10% of the nursing assistants missing injuries requiring the missing of less than one week of workdays while another 10% of the nursing assistants missed one to two weeks of workdays due to the pain (see Fig. 3).

**Fig. 4.**
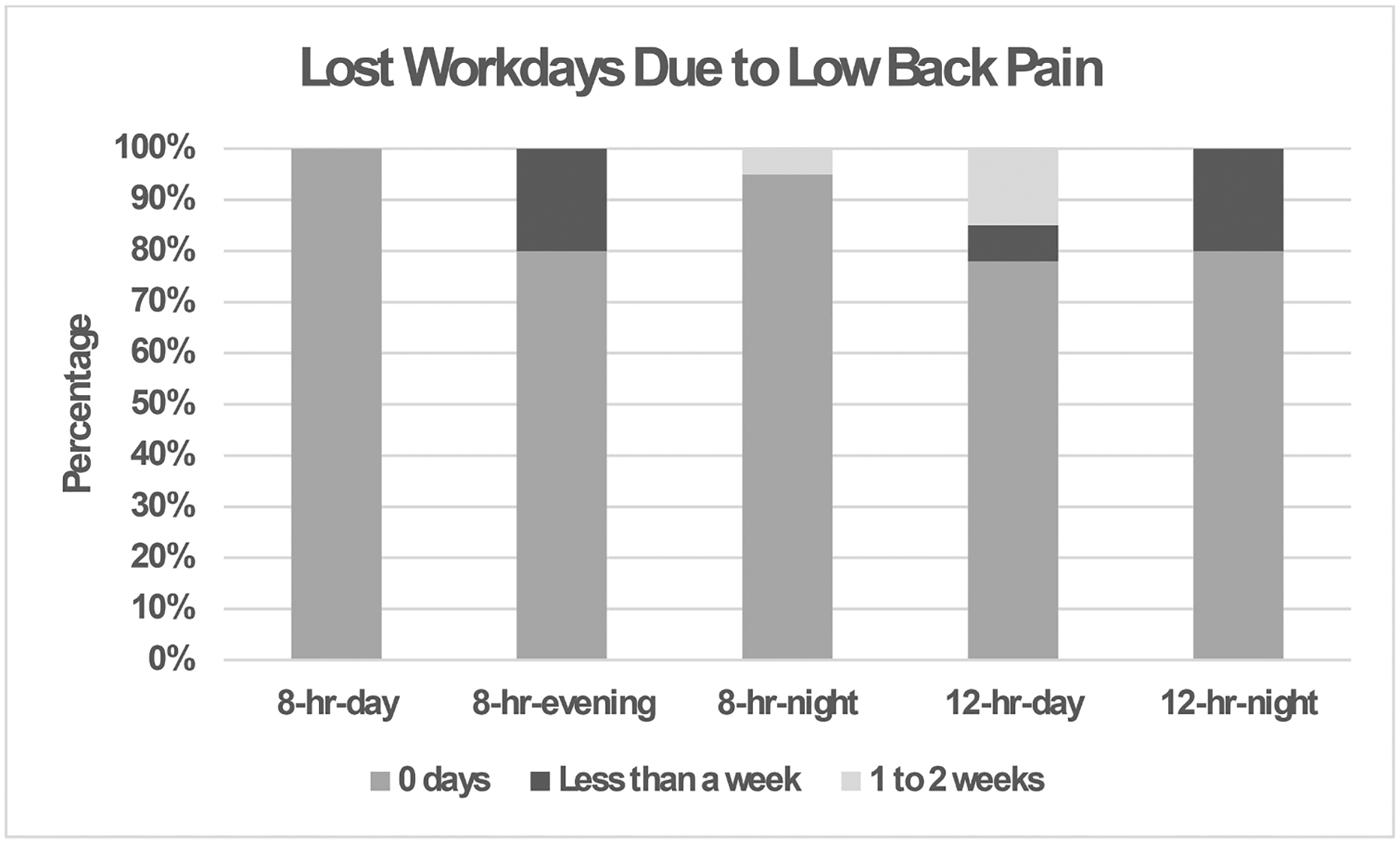
Percentage of nursing assistants who lost workdays due to low back pain (*p* = 0.26) in the past year for each shift (8-hr-day, 8-hr-evening, 8-hr-night, 12-hr-day, and 12-hr-night).

Lower back pain had the most lost workday injuries for the nursing assistants (Fig. 4). No significant difference was found among the nursing assistants for current low back pain (*P* = 0.23). However, similar to the low back pain in the past year, all shifts had a high prevalence of low back pain. In Fig. 4, 8-hr-evening and 8-hr-night shifts had more low back pain than other shifts (trend but insignificant).

**Fig. 5.**
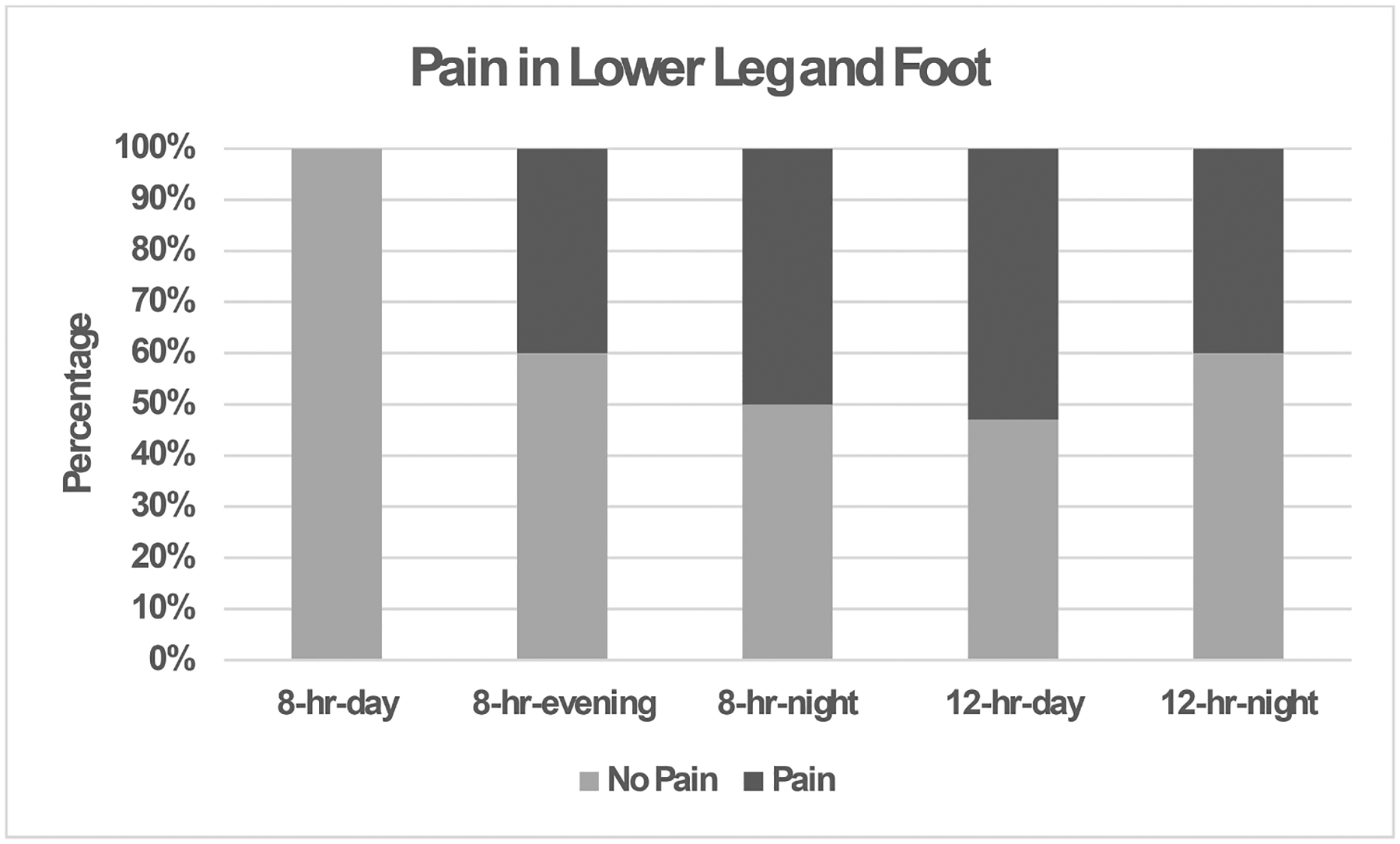
Percentage frequency of pain in the lower leg and foot for nursing assistants for each of the shifts (8-hr-day, 8-hr-evening, 8-hr-night, 12-hr-day, and 12-hr-night) in the past year (*p* = 0.04).

The pain was lower for 12-hr than 8-hr shifts (Fig. 5). Nursing assistants reported no pain on the 12-hr-day shift for the lower leg/foot and hand/wrist. The difference in the percentage of nursing assistants’ lower leg/foot pain was substantial for each shift in the past year, except for the 12-hr-day shift. The pain levels for the 12-hr shifts were 40% and 60% for 8-hr shifts, respectively, for the shoulder and neck areas in the past year. The 8-hr shifts had about 50%, indicating the presence of shoulder and neck pain. The 12-hr-night shift had the highest prevalence of hip pain, with 30% of nursing assistants reporting moderate to severe hip pain. Nearly half of the assistants on the 8-hr shifts had moderate to severe lower back pain.

## 4. Discussion

Each of the five long-term care facilities had both 12-hour and 8-hour shifts to compare different workload intensities. Previous studies focused on the turnover and retention of existing staff in addressing the workforce issue. In contrast, this study has emphasized the impact of the work shift on **physical demands and activity of** existing staff **as well as the** musculoskeletal injuries among nursing assistants.

Each of the long-term care facilities had the exact same shift schedules which comprised three 8-hour shifts or two 12-hour shifts. Overall, the workers on 8-hr and 12-hr shifts work *approximately* equal amounts of time a week (e.g. 40 h for 8-hour and 36 h for 12-hour shifts). The 12-hour shift workers have extended working hours **each day** compared to 8-hour shift workers but work fewer hours per week. Further, this study shows that 12-hr shifts have workload activities less than 8-hr shift caregivers. The day shift nursing assistants were on their feet longer than the evening and the night shift nursing assistants. The differences in physical activity between the facilities were insignificant. Only 10% of the shift for the day shift was spent sitting, mainly at lunchtime, with the rest of the time on their feet, either walking or standing.

For this study, the 12-hr shifts had similar tasks to 8-hr shifts, with shift changes happening at the end of the shifts. For example, the 12-hr-day shifts had similar tasks as those for the 8-hr-day shifts, but 12-hr-day nursing assistants assist the residents at dinner and afternoon naps. The 12-hr-night shift requires the same tasks as the 8-hr-night shift such as getting residents showered and ready for bed and nightly medicines. Residents are routinely checked every two hours, no matter what shift, especially if they have special needs or are handicapped.

### 4.1. Activity levels

The most apparent data was that the 8-hr-day shift was the most energy-consuming, mainly walking and less sitting, compared to other shifts. This result was not unexpected as most activities in the nursing home are conducted during the day (i.e., waking up, cleaning, showering, etc.). In addition, 8-hr-day shift nursing assistants need a fast pace to finish the job; therefore, the body likely increases heart rate and consumes more energy to adapt to the workload. The 12-hr shifts had less energy consumption hourly than the 8-hour shifts. This indicates that although the nursing assistants work extended hours in the 12-hour shifts, the workload was potentially relatively dispersed hourly and may reflect a less active nighttime period. The bottom line is that the workload for 8-hour and 12-hour shifts is significantly different.

JÄRvelin-Pasanen and associates found some differences between the shifts, which may indicate that the more flexible organization of work duties possible during extended work shifts (12-hour) allows for better regulation of physical activity. An interpretation could be that nursing assistants on the 12-hour shift appear to be able to regulate their hourly activity rate better. This conclusion was supported by a study from the National Institute of Occupational Safety and Health testing the heart rate variability between 8-hour and 12-hour shifts in nursing work (JÄRvelin-Pasanen et al., 2013).

Further evaluations of the physical work demands for the nursing assistants on 12-hour shifts may be overall more demanding as fatigue builds up over the shift. Berastegui et al. reported that the feeling of tiredness and fatigue was often greater for 12-hour shifts than for 8-hour shifts (Berastegui et al., 2020). Within this study, the 12-hour shift nursing assistants had nearly the same time spent standing (5 to 5.5 h) but had less time sitting. These breakdowns in walking, sitting, and standing are likely linked to the tasks and workflow requirements as the 12-hour shifts incorporate the 8-hour shift for longer durations. Walking and standing are only one part of the physical activity. Patient handling is another major contributor to physical activity and fatigue, which was not measured in the current study.

**Fig. 6.**
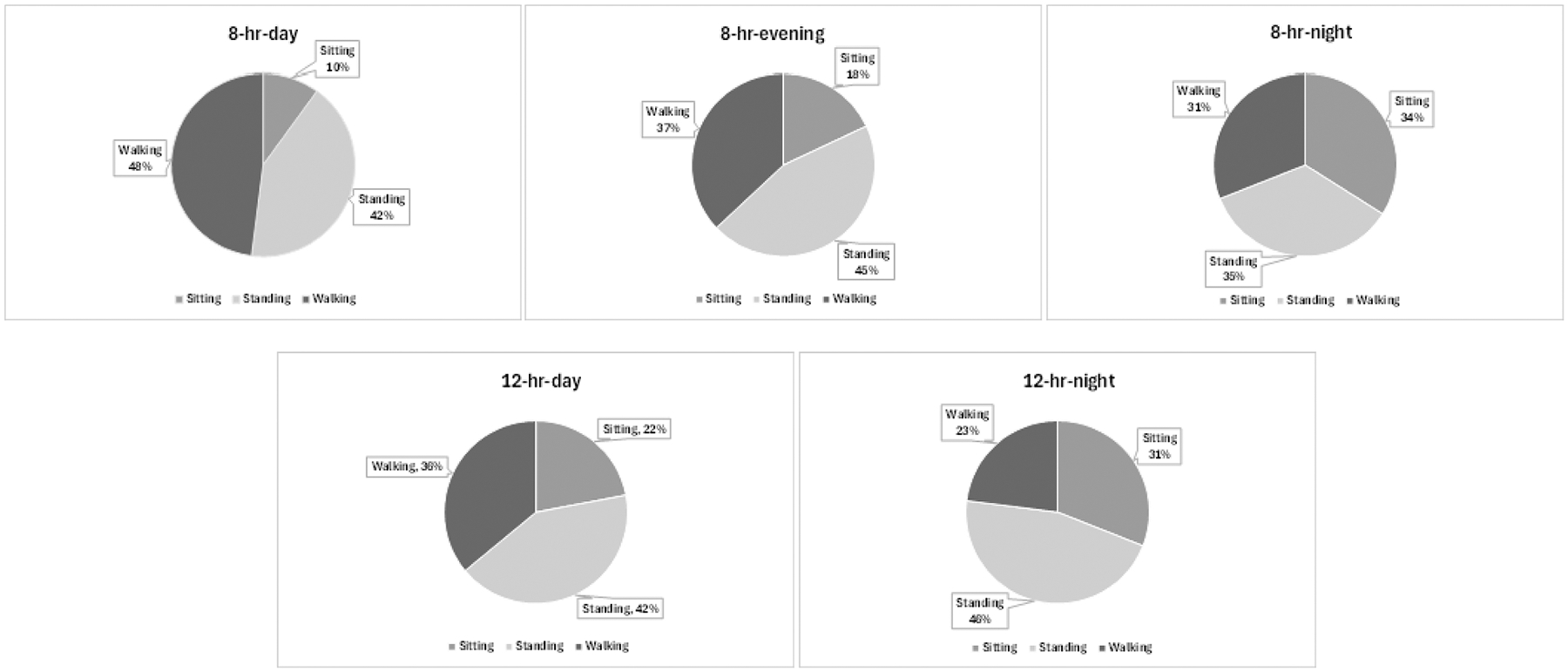
Percentage of time spent sitting, standing, and walking for the different shifts (8-hr-day, 8-hr-evening, 8-hr-night, 12-hr-day, and 12-hr-night).

The four extra hours for the extended shift (12 h) and the appropriate level of physical demand in elderly care facilities need to be analyzed during similar time periods (Fig. 6). The day shifts spent more time walking (*P* = 0.001), while the night shifts spent more time sitting (*P* = 0.03). Physical activity (e.g., more standing and walking) may be the precursor for the high levels of musculoskeletal pain and injuries found in nursing assistants, especially in the lower extremities.

### 4.2. Posture

The REBA score calculated in this study was revised from the traditional REBA score calculation by adding computer-use items designed and validated with a study in hospital settings (Ayvaz et al., 2023). The results showed that the 8-hour (shift night) had the highest score, indicating more awkward postures and more weight handling. The higher upper body index may reflect the additional tasks commonly performed by the nursing assistants on this shift, such as bathing, repositioning a resident in bed, changing clothing, and transferring a resident from bed to chair or toilet (Melles et al., 2024; Nelson et al., 2003). As in the research of Janowitz and associates (2006), the average individual upper and lower extremities scores were 9.0 and 6.6 for 178 hospital nurses compared to 7.0 and 6.8 (highest number among the shifts, 8-hour shift “night”) in the current study (Janowitz et al., 2006).

The revised REBA score showed a medium-high strain of postures for nursing assistants on each shift. It also indicated that improper postures might be adopted by the nursing assistants on an 8-hour shift for the upper extremity. The current study also sheds light on a future direction for investigating high-strain postures with force exerted by nursing assistants when performing patient-handling tasks.

The posture of the nursing assistants was observed on an average of every five-minute interval and then recorded for 8 or 12 h, depending on what shift duration the nursing assistants worked on. Nearly 20% of all of the observations for the nursing assistants had poor posture, as well as 44% of the observations for the right elbow (flex<60, ext>100) for the 8-hour “night” shift. These poor upper extremity postures may have resulted in the most interaction with the residents for an 8-hr-day shift. Moreover, the 12-hour shift had more high-risk wrist postures for the right arm. While there was no significant difference among the shifts for trunk posture, a trend was observed that the 8-hr-night shift involved more trunk flexion, where nursing assistants routinely flexed forwards more than 60°. This may have resulted from more patient handling tasks that are unassisted. Kozak (2017) used a quantitative method to assess the awkward back posture (inclination of ≥60°), which accounted for 22% of the working time, with tasks such as making the bed and cleaning (16%) being responsible for this awkward posture (Kozak et al., 2017). Night shift nursing assistants are not likely to do these activities but indicate how specific tasks impact posture. Further research is needed to understand what is driving the poor trunk posture of night shift nursing assistants.

The percentage in Table 5 was the awkward postures observed out of the total 96 (8-hour shift) or 144 (12-hour shift) observations. For the awkward trunk posture, the times of trunk bending over 60° for each shift were similar. During the day shifts, the composite tasks of making beds, providing basic care, and cleaning demonstrated that postures involved bending over 60°. Therefore, examining the trunk postures for other shifts, particularly the night shifts, and other at-risk joints should be expanded.

### 4.3. Injuries and pain

There are no differences between facilities on work absences. Most of the nursing assistants had musculoskeletal pain in multiple body regions. The 8-hr-night nursing assistants had more hand and wrist pain and a higher prevalence of knee pain. In addition, the 8-hr-night shift had the most lost workdays due to foot and ankle pain. The 8-hr-night shift had the most leg and foot pain in the past year.

Musculoskeletal pain was more prevalent for nursing assistants on the 8-hour shift than on the 12-hour shift. Although some of the symptoms for the 12-hour shift were more severe than the 8-hour shift, the overall prevalence was lower. For example, the 12-hour shift had hand and wrist pain; about 30% and 10% reported moderate to severe pain. On the other hand, 60% of the nursing assistants on the 8-hour “night” shift reported having moderate pain.

There was no significant difference in low back pain. Still, the 8-hour “night” shift had the highest prevalence of 80% of nursing assistants on that shift reported having moderate to high levels of low back pain last year; the 12-hour shifts had lower prevalence at 30% of nursing assistants reporting moderate to a high level of low back pain in the past year. These pain levels were relatively high compared to the previously reported prevalence of lower back pain (Davis & Kotowski, 2015). Studies done for musculoskeletal disorders showed that the prevalence of lower back pain for nursing assistants was from 45% to 76% in the previous year. Still, lower back pain had the highest prevalence among the pain in different body regions (Lee et al., 2015; Lin et al., 2020). Nursing assistants have often been found to be a vulnerable population regarding low back pain and injuries compared to nurses (Fuortes et al., 1994; Sampath et al., 2019). A high prevalence of lower back pain in the present study indicated lower back pain is severely affecting the nursing assistants’ population. Others have found low back pain is not the only pain being suffered by nursing assistants in long-term care facilities (Dong et al., 2019).

### 4.4. Limitations

Further analysis of the workers’ movement could have been done such as an explanation of which movement belongs to which type of work. Although differences in physical activities, body postures, and MSD symptoms between shifts were observed, the mechanism by which shift work affects the development of MSD was not examined. The study provides some interesting trends that should be investigated **to help us** to understand the higher rates of MSDs for nursing assistants in long-term health facilities.

This study had limited nursing homes (five) and 54 workers, so there was limited coverage of different facility cultures and processes. Individual long-term care environments and management cultures may differ and could impact the trends in the current study. The current study only investigated five facilities in the greater Cincinnati area. For example, a long-term care facility in a rural area could be a different situation due to nursing assistants shortage, shift assignment, and resident characteristics (physical demand).

The study faced limitations in quantifying patient handling activities, specifically the absence of detailed measurements regarding the frequency of patient handling per hour and the weight or exertion force applied during such activities. This lack of precise data constrains the accuracy of physical workload assessments. The patient’s weight is crucial in estimating the physical demands of nursing assistants during lifting or transferring tasks. Although the force exertion required for calculating the Rapid Entire Body Assessment (REBA) score was estimated, this approach may lead to underestimating the physical strain. Furthermore, the observational methodology employed to document patient handling tasks was insufficiently detailed and lacking in specificity and clarity regarding the locations and nuances of the tasks. This deficiency hindered accurate estimations of force exertion. It impeded the comprehensive understanding of the postures nursing assistants assumed during weight or patient handling, thereby complicating the assessment of their physical workload.

The characteristics of the demographic of the nursing assistants could lead to bias. The nursing assistants who participated in the study showed that half of the subjects were Caucasian (50.8%), nearly half were African American (44.3%), and a small portion were Hispanic (1.6%) and Asian (3.3%). The 8-hr-night shift was relatively younger, averaging 26.5 years old, employed less time with their current employer (14.9 months), and less experienced (35.1 months). In addition, the BMI of the nursing assistants showed that they were all over-weight, and night shifts, both 8 and 12 h, were more obese. These demographic characteristics could have influenced the postures and physical activities.

### 4.5. Conclusion

Significant differences exist in physical workload among shifts, with the 12-hour shift having the least energy expenditure per hour despite its longer duration. The 8-hour shifts have more concerns, with this study’s analysis of posture and activity having 8 of the 12 highest risk factors. The 8-hr-night shift posed the highest risk for musculoskeletal disorders, with night shift nursing assistants exhibiting more musculoskeletal symptoms than day shift assistants despite a lower workload. This highlights the need to evaluate specific tasks, such as assisting clients to bed, as potential high-risk activities. The findings emphasize the importance of providing adequate training on proper posture and working habits to reduce the risk of musculoskeletal disorders among nursing assistants.

### 4.6. Highlights

There was a significant difference in physical workload among the shifts. Although 12-hour shift work was longer, the workload was dispersed throughout the shift with the least energy expenditure per hour. Postures adopted by nursing assistants were different for each of the shifts.

The 8-hr-night shift had the highest musculoskeletal disorder risk compared to other shifts. The nursing assistants working 8-hr-day shifts had less prevalence of musculoskeletal symptoms than 8-hr-evening and 8-hr-night shift nursing assistants even though they had a higher workload. The high musculoskeletal injuries demonstrate the need to evaluate specific tasks, such as helping clients go to **bed and repositioning**, as a possible higher risk to workers.

Based on this investigation, sufficient training on proper posture and working habits was necessary for nursing assistants to decrease the risk of developing musculoskeletal disorders. For example, nursing assistants **need to** raise the bed when routinely checking on patients, which could significantly reduce the bending forward posture above 60°.

## Figures and Tables

**Table 1:** Summary of demographics for the subjects who participated in the study as a function of shift.

	Shift									
	8-hr-day		8-hr-evening		8-hr-night		12-hr-day		12-hr-evening	
	Mean	Std. Dev.	Mean	Std. Dev.	Mean	Std. Dev.	Mean	Std. Dev.	Mean	Std. Dev.
**Number of Nursing Assistants (N)**	12		12		10		10		10	
**Age (years)**	34.5	13.5	27.0	4.3	32.5	12.5	34.7	11.3	42.1	10.2
**Standing Height (cm)**	165.4	13.0	162.8	6.4	165.6	5.3	164.8	3.8	167.1	5.1
**Body Weight (kg)**	76.1	17.2	79.2	18.9	88.1	20.3	70.2	11.1	86.2	17.9
**Time with Current Employer (months)**	90.3	88.0	15.2	14.3	48.2	61.9	25.9	23.6	45.2	30.2

**Table 2:** Summary of the dependent variables, measurements, and statistical analysis methods.

Dependent VariableCategory	Measurement or Instrument	Dependent Variables	Statistical Analysis
Physical Demands	ActivPAL	Energy Expenditure Time spent on Standing, Sitting, Walking Total Steps/Hour	Descriptive Analysis
	Rapid Entire Body Assessment (REBA Checklist)	Upper Body and Lower Body REBA Score	One-Way ANOVA
		Hourly Awkward Posture Frequencies	Kruskal-Wallis Test
Body Discomfort	Survey	Current Pain level for body regions	Fisher Exact Test
Lost day of work	Survey	Current Pain level for body regions	Fisher Exact Test

**Table 3:** Physical activity level for each of the shift (8-hr-day, 8-hr-evening, 8-hr-night, 12-hr-day, and 12-hr-night) including energy expenditure, total steps, and time spent standing, sitting, and walking.

Shift	Energy Expenditure (MET)	Total Steps Taken (steps/hr)	Total Time Standing (% of shift)	Total Time Sitting (% of shift)	Total Time Walking (% of shift)
8-hr-day	2.07	1704	41.7%	9.8%	48.5%
8-hr-evening	1.94	1356	45.1%	17.6%	37.3%
8-hr-night	1.76	1101	34.6%	34.6%	30.8%
12-hr-day	1.84	1388	42.4%	21.6%	36.0%
12-hr-night	1.62	763	45.8%	31.3%	23.1%

**Table 4:** Average counts/hour of different joint angles range for severe posture by shifts (8-hr-day, 8-hr-evening, 8-hr-night, 12-hr-day, and 12-hr-night). Bold p-values indicate significant at *p* < 0.05.

Joints	Angles	8-hr-day	8-hr-evening	8-hr-night	12-hr-day	12-hr-evening	P-Value
Neck	flex>20, ext>20	1.85	1.15	1.04	1.03	1.48	**0.02**
Left Shoulder	flex>45, ext>20	1.22	2.15	2.39	1.58	2.03	**0.03**
Left Wrist	flex15–22, flex/ext >22	2.85	4.23	3.49	4.19	3.72	**0.02**
Right Elbow	flex<60, ext>100	3.11	3.84	5.3	4.73	4.78	**0.03**
Right Shoulder	flex>45, ext>20	1.28	1.77	2.85	2.02	2.13	**0.02**
Right Wrist	flex15–22, flex/ext >22	2.71	3.8	3.9	4.17	4.13	**0.03**
Trunk	flex>60 ext>20	1.29	1.63	2.09	1.51	1.48	0.52

**Table 5:** Percentage of awkward posture observations for each of the shift (8-hr-day, 8-hr-evening, 8-hr-night, 12-hr-day, and 12-hr-night).

Joints	Angles	8-hr-day	8-hr-evening	8-hr-night	12-hr-day	12-hr-evening
Neck	flex>20, ext>20	15.4%	9.6%	8.7%	8.6%	12.3%
Left Shoulder	flex>45, ext>20	10.2%	17.9%	19.9%	13.2%	16.9%
Left Wrist	flex15–22, flex/ext >22	23.8%	35.3%	29.1%	34.9%	31.0%
Right Elbow	flex<60, ext>100	25.9%	32.0%	44.2%	39.4%	39.8%
Right Shoulder	flex>45, ext>20	10.7%	14.8%	23.8%	16.8%	17.8%
Right Wrist	flex15–22, flex/ext >22	22.6%	31.7%	32.5%	34.8%	34.4%
Trunk	flex>60 ext>20	10.8%	13.6%	17.4%	12.6%	12.3%
